# Novel variant alters splicing of *TGFB2* in family with features of Loeys-Dietz syndrome

**DOI:** 10.3389/fgene.2024.1435734

**Published:** 2024-12-16

**Authors:** Emily R. Gordon, Stephanie A. Felker, Tanner F. Coleman, Nadiya Sosonkina, Jada Pugh, Meagan E. Cochran, Anna C. E. Hurst, Sara J. Cooper

**Affiliations:** ^1^ HudsonAlpha Institute for Biotechnology, Huntsville, AL, United States; ^2^ Department of Genetics, University of Alabama at Birmingham, Birmingham, AL, United States; ^3^ Smith Family Clinic for Genomic Medicine, Huntsville, AL, United States; ^4^ Molecular and Human Genetics, Baylor College of Medicine, Houston, TX, United States

**Keywords:** splicing, Loeys-Dietz, TGFB2, noncoding variation, WGS, connective tissue disorder, SpliceAI, clinical genomics

## Abstract

Loeys-Dietz syndrome (LDS) is a connective tissue disorder representing a wide spectrum of phenotypes, ranging from isolated thoracic aortic aneurysm or dissection to a more severe syndromic presentation with multisystemic involvement. Significant clinical variability has been noted for both related and unrelated individuals with the same pathogenic variant. We report a family of five affected individuals with notable phenotypic variability who appear to have two distinct molecular causes of LDS, one attributable to a missense variant in *TGFBR2* and the other an intronic variant 6 bp upstream from a splice junction in *TGFB2*. We tested the functional impacts of the variant identified in the proband alongside other variants in the region reported in ClinVar using a splice reporter system, which resulted in non-canonical splicing products for several variants including the proband. Molecular validation of the splicing products suggests that the *TGFB2* variants tested impact splicing by reducing efficiency of the canonical acceptor in favor of an alternate acceptor within the exon. These data combined with clinical phenotypes and segregation of the variant with disease support the conclusion that this intronic *TGFB2* variant may cause LDS in this patient and her mother. These analyses demonstrate that underappreciated intronic variants that alter splicing can be relevant for clinical phenotypes of connective tissue disease. This case highlights the importance of prompt familial cascade testing, clinical evaluation with detailed dysmorphology exam, comprehensive genetic testing, and collaboration between clinicians and scientists to characterize variants of uncertain significance to properly assess risk in LDS patients.

## 1 Introduction

Loeys-Dietz syndrome (LDS) is a rare connective tissue disorder with a prevalence of 1 in 100,000 individuals (Loughborough et al., 2018). Affected individuals present with a wide spectrum of phenotypes, ranging from isolated thoracic aortic aneurysm or dissection to a severe syndromic presentation with multisystemic involvement. Due to the incidence of aortic aneurysm and dissection, the life expectancy of an individual with LDS averages 37 years old, although the range is highly variable depending on severity (Loeys et al., 2006; Mazzella et al., 2017). LDS diagnosis typically requires angiograms, cardiac imaging, basic family history, medical exams, and genetic testing for molecular diagnosis.

LDS results from pathogenic heterozygous loss-of-function variation in one of several TGF-beta pathway members: *SMAD2*, *SMAD3*, *TGFB2*, *TGFB3*, *TGFBR1*, or *TGFBR2*. The majority of cases (75%) are the result of *de novo* mutations in one of these six genes and the remainder are inherited in an autosomal dominant manner. Significant clinical variability has been noted for both related and unrelated individuals with the same pathogenic variant, and there is documented reduced penetrance, so even when a LDS-causing variant is inherited, parents may not show clinical features of the condition (Loeys and Dietz, 2008).

While the pathway affected in LDS patients is well-described, only about half of LDS patients have a molecular diagnosis (Renner et al., 2019). Inability to provide a genetic diagnosis results from a variety of challenges including a limited ability to predict the impact of non-coding, intronic, and synonymous variants. Advancements in whole genome sequencing (WGS) have significantly shortened the diagnostic odyssey and increased the frequency of molecular diagnosis in rare diseases cases, including LDS. In LDS, genetic diagnosis improves management of patients because treatment and imaging recommendations vary depending on the specific cause (Ikonomidis, 2017; MacCarrick et al., 2014; Krohg-Sørensen et al., 2017; Isselbacher et al., 2022). In our study, we explore the function of variants that could alter splicing in LDS-associated genes. We use SpliceAI predictions (Jaganathan et al., 2019) to prioritize variants that may alter splicing (Hirschi et al., 2024). Our study characterizes the functional impact of intronic variants of the LDS-associated gene *TGFB2* including one observed in a unique family with an LDS diagnosis and other individuals with features of LDS.

We describe a family with three confirmed LDS diagnoses explained by a missense variant in *TGFBR2.* Additional family members were identified with features of LDS, but genetic testing ruled out the familial *TGFBR2* variant. Instead, genome sequencing revealed an intronic variant in *TGFB2* [NM_003238.6 (TGFB2):c.755-6T>C], another LDS-associated gene. We used a splice reporter assay to characterize this variant which occurs 6bp upstream of the splice acceptor in exon 5 of *TGFB2*. Our data provides new evidence that this splice-altering allele may lead to LDS-like features in this unique family that appears to have two distinct variants in LDS-associated genes resulting in similar LDS-associated phenotypes. This case demonstrates the value of thorough genetic evaluation and emphasizes the potential impact of non-coding sequences to further our understanding of LDS.

## 2 Methods

### 2.1 Clinical evaluation of connective tissue disorder

Patients were evaluated for features of connective tissue disorders based on the standard of care process. Evaluation by a medical geneticist included a physical exam, a 92-gene connective tissue disorder panel, and targeted sequencing as described in detail in the Results section. Full details of the clinical evaluation are available in Supplementary Material S3.

### 2.2 Genome sequencing

Whole Genome Sequencing was performed by HudsonAlpha Clinical Services Lab, LLC (“CSL”) (CLIA: #01D2086581; CAP: #8051488; Lab Director: Ghunwa Nakouzi, PhD), using the Illumina NovaSeq 6000 sequencing platform. DNA was checked for quality and concentration and sonicated to a specific fragment size. After size selection, DNA fragments were ligated to a unique pair of Illumina flow cell-specific index adapters. No amplification of the library was performed. The prepared library was quality-checked for adequate yield with fluorescence methods and quantitative polymerase chain reaction (qPCR) and for appropriate fragment size distribution using microfluidics-based platforms. The library was pooled with similar type libraries, denatured, and loaded onto Illumina NovaSeq 6000 flow cells and sequenced using standard Illumina reagents and protocols.

### 2.3 Data processing and quality control

Genome sequencing data were collected using Illumina sequencing and reads were aligned to the human reference GRCh38. Quality control metrics were calculated, with low quality reads being removed. Variant calling proceeded using unique, high quality reads. See Supplementary Material S3 for additional details.

### 2.4 Genomic analysis

Sequence variants were analyzed using a custom software analysis application called Codicem (v. 5.7.4) for interpretation. See Supplementary Material S3 for filtering restrictions and details. Variants were classified based on the ACMG/AMP guidelines (Richards et al., 2015). All primary, actionable secondary, and complex indel variants were confirmed by an orthogonal technology (Sanger (dideoxy) sequencing). Other reported variants were assessed by a validated algorithm and orthogonally confirmed when metrics indicated a possible false positive (Holt et al., 2021) Patient-specific orthogonal confirmation details are available upon request.

### 2.5 Analysis of variants submitted to ClinVar database

Variants near the proband variant were acquired from ClinVar (July 2023), and intronic and synonymous variants with no predicted amino acid or termination change were annotated using Ensemble’s Variant Effect Predictor (VEP, McLaren et al., 2016) and the SpliceAI (Jaganathan et al., 2019) web server (https://spliceailookup.broadinstitute.org/) to predict the effect of each variant on splicing within 500 bp of the variant. We used an additional computational predictor, Combined Annotation Dependent Depletion (CADD) score, to further filter variants for consideration (Rentzsch et al., 2021). One likely pathogenic positive control variant [NM_003238.6 (TGFB2):c.755-5_755-2delinsG] and two common negative control variants (NM_003238.6 (TGFB2): c.755-5dup and NM_003238.6 (TGFB2):c.747A>G (p.Arg249 = ) were selected. An additional test variant classified as Likely Benign [NM_003238.6 (TGFB2):c.644-18T>A] was also selected for analysis, due to positive computational predictors. The ClinVar submitters of these variants were contacted for phenotype information, when available.

### 2.6 Molecular validation

We used the pSpliceExpress vector system (Addgene #32485) and methods previously established by (Kishore et al., 2008) to conduct a splice reporter assay (Supplementary Material S3). Sequences were synthesized as gBlocks HiFi gene fragments (IDT). The gBlock harbored the three exon-intron-exon-intron-exon junctions of interest along with flanking 200 base pairs of introns and attB1 sites. Six sequences were tested: reference (GRCh38), NM_003238.6:c.755-6T>C (proband), and four sequences reported in ClinVar not previously characterized by splice reporter (Table 1). Each of these variants falls in the intronic sequence near the variant NM_003238.6:c.755-6T>C (Supplementary Table S1). Cloned and purified plasmids were sequence verified with whole plasmid sequencing using Oxford Nanopore Technology (Plasmidsaurus, Eugene, OR).

**TABLE 1:** Variant table including SpliceAI scores.

HGVS DNA reference	Variant type	ClinVar ID	ACMG classification	gnomAD v3.1.2 allele count	CADD score	SpliceAI ΔScore acceptor gain	SpliceAI ΔScore acceptor loss	SpliceAI ΔScore donor gain	SpliceAI ΔScore donor loss
NM_003238.6 (TGFB2): c.755-6T>C	SNV	2,017,460	likely benign	0	19.78	0.03	0.1	0	0
NM_003238.6 (TGFB2): c.755-5_755-2delinsG	Deletion	528,887	likely pathogenic	0	N/A	0.14	0.91	0	0
NM_003238.6 (TGFB2): c.755-5dup	Duplication	2,165,076	benign	7	14.51	0	0	0.03	0
NM_003238.6 (TGFB2): c.747A>G (p.Arg249 = )	SNV	387,890	likely benign	45	14.98	0	0	0.02	0.18
NM_003238.6 (TGFB2): c.644-18T>A	SNV	511,039	likely benign	0	22.3	0.02	0.57	0	0

HEK293T cells were seeded at a density of 5 × 10^5^ cells per well of a 6 well plate 24 h prior to transfection in 2 mL of media. Transfection into HEK293T cells took place per well using 1 μg of reporter plasmid, 12.5 μL Lipofectamine 2000 (ThermoFisher #11668019), 10 μL Plus reagent (ThermoFisher #11514015), and 400 μL OptiMem (ThermoFisher #11058021). Twenty-four hours following transfection, the cells were harvested, and RNA was extracted with the Norgen Total RNA Extraction Kit following the manufacturer’s protocol including DNase treatment followed by freezing at −80°C (Norgen #37500 and #25710).

Two step RT-PCR using 300 ng of RNA was performed. cDNA was made using random hexamer (50 ng) primed Superscript first-strand synthesis system (Invitrogen #11904-018) and frozen at −20°C. Thirty nanograms of template cDNA was amplified by PCR using primer sequences in the rat insulin exons (RIE2 or RIE3) (5′-CCT​GCT​CAT​CCT​CTG​GGA​GC-3′ and 5′- AGG​TCT​GAA​GGT​CAC​GGG​CC-3′) using Platinum SuperFI PCR MasterMix following manufacturer’s protocol for a 20 μL reaction (Invitrogen #12368010). PCR conditions were as follows: initial denaturation 98°C for 30 s; 30 cycles of 98°C for 10 s, 66°C for 10 s, and 72°C for 1 min; and a final extension at 72°C for 5 min. PCR products were frozen at −20°C. Products were gel purified using 2% agarose gel electrophoresis with SYBR Green I gel electrophoresis dye per manufacturer’s recommendations (Sigma #S9430). Gels were visualized (Bio-Rad ChemiDoc Imaging System) and bands were manually cut out. DNA was extracted from individual bands (Zymo #D4007) and sent for Sanger sequencing (Molecular Cloning Laboratory) (Supplemental Table S2). Intensity of gel bands was quantified using ImageJ 1.53 K (http://imagej.nih.gov/ij), background signal was normalized, and fraction of total intensity for each band was calculated. Graphs and statistics were generated using GraphPad Prism (v10). Significance was determined with an unpaired parametric *t*-test. All steps were performed sequentially in quadruplicate unless otherwise noted.

## 3 Results

### 3.1 Clinical presentation and family history: NM_003238.6 (TGFBR2):c.412T>G p.Cys138GLy

The first individual (II:3, Figure 1A) evaluated at the Smith Family Clinic for Genomic Medicine in Huntsville, AL was a 53-year-old male evaluated for possible connective tissue disorders due to bicuspid aortic valve and thoracic aorta aneurysm of 4.6 cm. His medical history was significant for microretrognathia, surgically repaired as a teenager, and kyphoscoliosis. On physical examination, he was noted to have malar flattening, atypical appearance of the chest (narrow upper thorax, sloping shoulders, S-shaped sternum), wide neck with webbing, slightly low-set ears with thick helix, and down slanting palpebral fissures. He did not meet the established clinical diagnostic criteria for Marfan syndrome (Loeys et al., 2010) (Supplementary Material S3).

**FIGURE 1:**
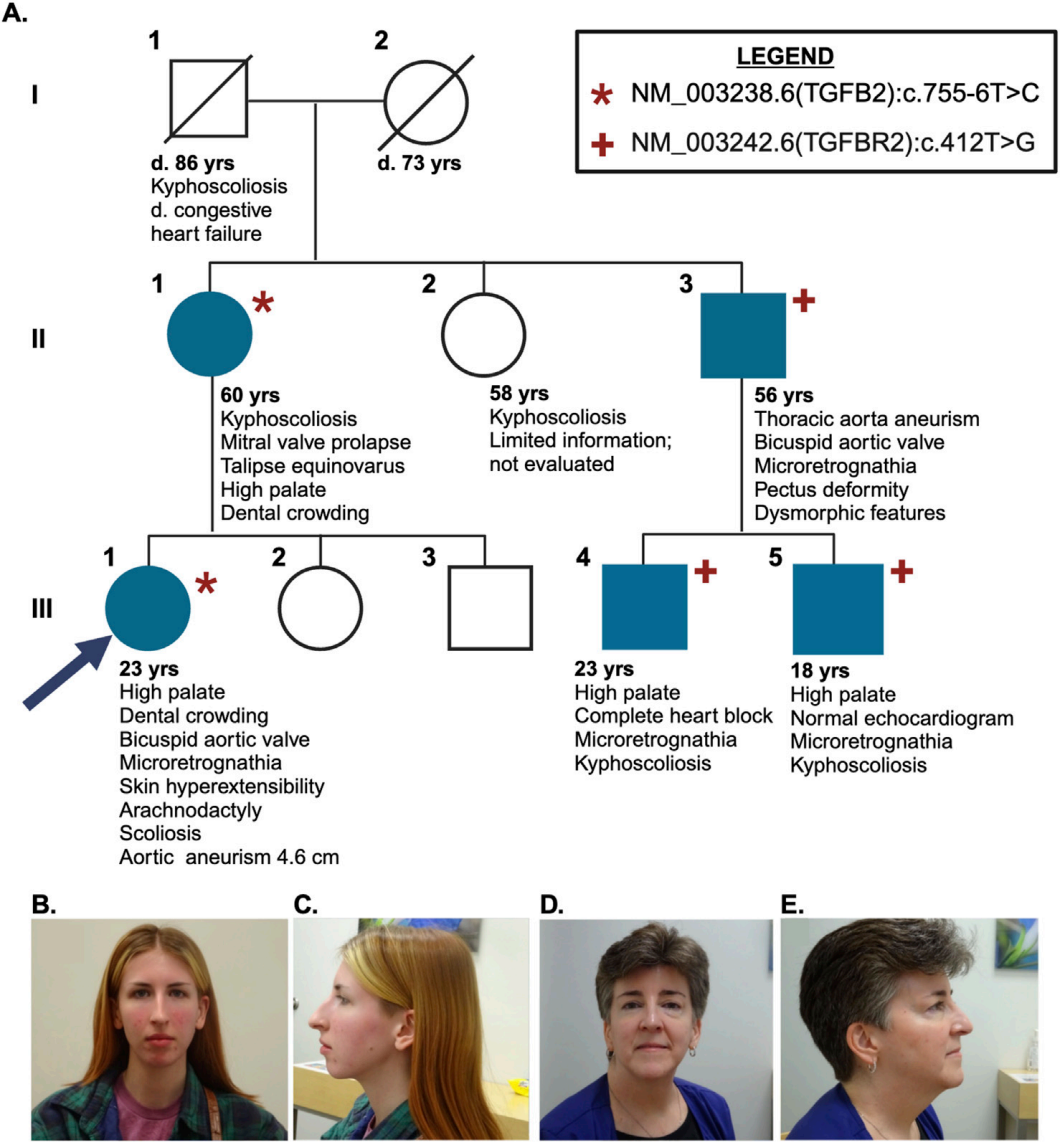
**(A)** Pedigree chart. The proband (III:1) is designated by an arrow. NM_003238.6 (TGFB2):c.T755-6T>C variant indicated in both proband (III:1) and affected mother (II:1) with the same variant indicated by an asterisk. Proband’s uncle (II:3) and similarly affected sons (III:4 and III:5) have a NM_003242.6 (TGFBR2):c.412T>G variant, indicated by plus sign. All affected individuals are shaded with teal. **(B)** Frontal photo of proband (III:1) **(C)** lateral photo of proband (III:1) **(D)** frontal photo of proband’s mother (II:1) **(E)** lateral photo of proband’s mother (II:1)

Individual II:3 underwent testing using a commercial panel of 92 connective tissue disorder genes, revealing a variant in *TGFBR2* (NM_003242.6 (TGFBR2):c.412T>G) classified as likely pathogenic by a commercial genetic testing lab with a proprietary variant calling software based on ACMG guidelines. No other pathogenic or likely pathogenic variants were reported in genes associated with any connective tissue disorders. He was subsequently diagnosed with LDS based on the presence of the likely pathogenic *TGFBR2* variant, his aortic root enlargement, and exclusion of connective tissue disorders with overlapping clinical features (Loeys and Dietz, 2008).

The patient’s similarly affected sons also harbor this *TGFBR2* variant, which was detected using targeted testing. The older son (III:4, Figure 1A) has a history of complete heart block at 10 months old, microretrognathia, kyphosis, and high palate. The younger son (III:5, Figure 1A) has microretrognathia, kyphosis, high palate, and normal echocardiogram. A diagnosis can be made in at-risk relatives of a person with a clinical diagnosis of LDS by identification of the pathogenic variant even if the other features are not yet present (Loeys and Dietz, 2008). The proband’s two sons (Individuals III:4 and III:5, Figure 1A) were diagnosed with Loeys-Dietz based on the presence of the diagnostic variant identified in their father.

The TGFBR2 variant NM_003242.6 (TGFBR2):c.412T>G p. Cys138Gly is present in gnomAD v3.1.2 at extremely low frequency (1/152,174, 0.0007%), and computational meta-predictors suggest that this variant is likely to be disruptive (CADD 26.3, REVEL 0.93) (Rentzsch, et al., 2021; Ioannidis et al., 2016). In addition to this proband and his sons, this variant has been reported as likely pathogenic in one additional patient with clinical features of TGFBR2-related disorders in the commercial laboratory’s internal database, and was reported to ClinVar as likely pathogenic by a single submitter, Labcorp Genetics (formerly Invitae), with assertion criteria (one star review status). Another variant impacting the same position and reported (ClinVarID 213915) (NM_003242.6 (TGFBR2):c.412T>C p. Cys183Arg) is further evidence supporting the likely pathogenic classification. This is a *de novo* variant in a patient with spontaneous cervical artery dissection and recurrent knee subluxations (Pezzini et al., 2011).

### 3.2 Identification of NM_003238.6 (TGFB2):c.755-6T>C

Proband III:1, the niece of II:3, also presented for evaluation at the Smith Family Clinic for Genomic Medicine (Figures 1A–C). Her medical history was significant for bicuspid aortic valve, microretrognathia requiring surgical repair, scoliosis requiring bracing, and dental crowding requiring extraction of 12 adult teeth. A recent echocardiogram revealed no aortic dilation or aneurysm. Upon physical exam, she was noted to have several features consistent with a connective tissue disorder. She has a long facial appearance, slightly downslanting palpebral fissures, very high-arched palate, and prominent sternum with inferior bilateral prominence at the rib attachments. Additionally, she has hyperextensible skin (4.5 cm neck, 1.5 cm volar surface of forearm), hypermobility of the shoulders and thumbs, and arachnodactyly (positive wrist and thumb sign) with long toes. She did not meet the established clinical diagnostic criteria for Marfan syndrome (Loeys et al., 2010).

The proband underwent targeted testing for the familial NM_003242.6 (TGFBR2):c.412T>G p. Cys138Gly variant but it was not observed. She subsequently underwent testing using the same commercial 92 gene panel for connective tissue disorders, which was also negative but did detect a likely benign variant NM_003238.6 (TGFB2):c.755-6T>C. This variant was submitted to ClinVar (RCV002835216.2) but not reported to the proband because of its likely benign classification at that time. The family then sought whole genome sequencing through the HudsonAlpha Clinical Services Lab (CLIA Number 01D2086581, CAP Number 8051488). The same TGFB2 variant [NM_003238.6 (TGFB2):c.755-6T>C] was identified, now known to be inherited from her mother (II:1; Figures 1A, D, E), confirmed by Sanger sequencing in both the proband and her mother (Supplemental Figure S1). Her mother is more mildly affected with mitral valve prolapse, kyphoscoliosis, congenital club foot, high palate with dental crowding, deviated nasal septum, and myopia. The mother also tested negative for the NM_003242.6 (TGFBR2):c.412T>G p. Cys138Gly variant identified in her brother. No other variants of interest were revealed upon analysis.

The NM_003238.6 (TGFB2):c.755-6T>C variant is located in the 3′ splice acceptor region between exons 4 and 5 of the TGFB2 protein. This variant is absent from gnomAD v3.1.2, and has mild predictive scores in SpliceAI (acceptor loss: 0.10, +6bp; acceptor gain: 0.03, +121 bp, CADD score: 19.78) indicating a low probability of this variant resulting in a 115 bp deletion from exon 5 compared to the canonical transcript. A formal diagnosis of LDS is not possible without the identification of a pathogenic variant in an associated gene (Loeys and Dietz, 2008). The TGFB2 variant [NM_003238.6 (TGFB2):c.755-6T>C] was classified as a variant of uncertain significance by the HudsonAlpha Clinical Services Lab (PM2_Supporting). The variant was thus reported to the family as a possible molecular diagnosis given the classification as a variant of uncertain significance, the overlap of the patient’s symptoms (presence of characteristic craniofacial, skeletal, and cutaneous features); segregation in the family; and exclusion of a diagnosis of overlapping clinical features with TGFB2-related disorders, and segregation with related phenotypes in the family.

### 3.3 Characterization of previously identified TGFB2 variants with predicted splicing impacts

Given that NM_003238.6 (TGFB2):c.755-6T>C is near the exon-intron border, we hypothesized that it could impact splicing. We also identified additional variants in the region using the ClinVar database and assessed their potential to alter splicing. These included a common benign and likely benign TGFB2 variants unlikely to cause disease as negative controls, a positive control variant classified as pathogenic based on known splicing impact, and a likely benign variant with positive predictors of pathogenicity (Table 1).

The positive control variant NM_003238.6 (TGFB2):c.755-5_755-2delinsG has a ClinVar (528,887) ACMG classification of likely pathogenic, is absent from gnomAD v3.1.2, has paired acceptor gain and loss of 0.14 and 0.91, according to SpliceAI, and predicts the same alternative splicing product as the proband.

Two negative control variants were chosen based on their increased allele count (AC) in gnomAD v3.1.2. The first is an intronic variant, NM_003238.6 (TGFB2):c.755-5dup, with a gnomAD AC of 7, CADD score of 14.51, and a predicted SpliceAI donor gain score of 0.03, 207 bp downstream of the variant. The second variant is exonic, NM_003238.6 (TGFB2):c.747A>G (p.Arg249 = ), with a gnomAD AC of 45, CADD score of 14.98, and a splice gain and loss scores of 0.02 and 0.18, respectively.

We also tested a likely benign variant identified in ClinVar, NM_003238.6 (TGFB2):c.644-18T>A that is absent from gnomAD v3.1.2, a CADD score of 22.3, and has a paired splice acceptor loss (SpliceAI score: 0.57, 18 bp downstream of variant) and gain (SpliceAI score 0.02, 2 bp downstream), which predicts an alternative splicing product with a 16 bp insertion in exon 4 of the transcript, suggesting a possible splicing impact.

### 3.4 Genomic and molecular analysis

**FIGURE 2:**
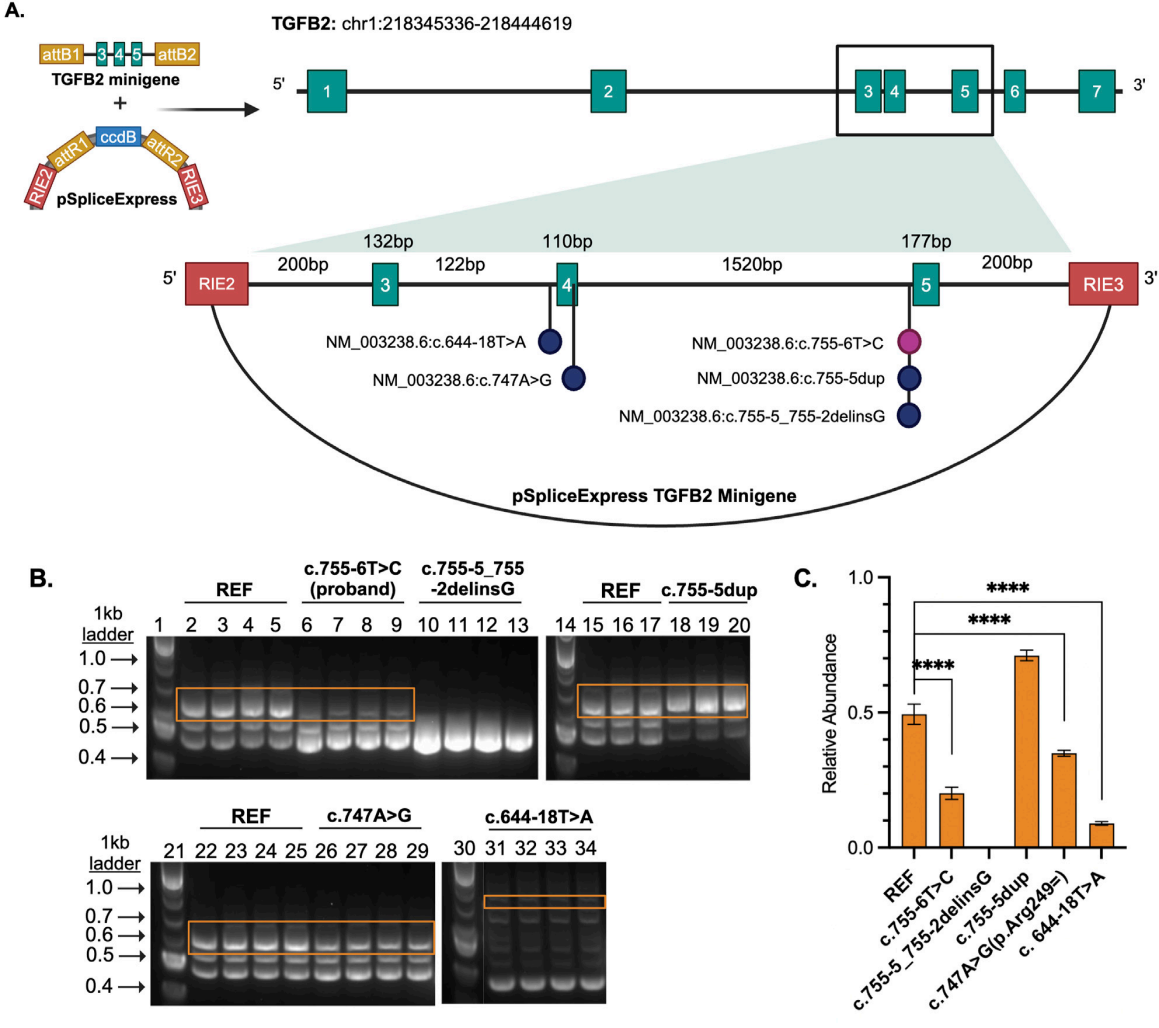
**(A)** TGFB2 minigene cloned into pSpliceExpress. RIE2 and RIE3 are rat insulin exons. Each vector contains either the reference, proband variant, or another variant of interest. **(B)** Gel electrophoresis of the RT-PCR products where lanes 1, 14, 21, and 30 are size markers (1 kb plus ladder), 2-5, 15-17, and 22-25 are reference (REF), 6-9 are proband NM_003238.6 (TGFB2):c.755-6T>C, 10-13 are likely pathogenic control NM_003238.6 (TGFB2):c.755-5_755-2delinsG, 18-20 are a benign NM_003238.6 (TGFB2):c.755-5dup, 26-28 are a likely benign NM_003238.6 (TGFB2):c.747A>G, and 31-34 a likely benign variant NM_003238.6 (TGFB2):c.644-18T>A. **(C)** Bar plot displays relative abundance of canonical splice product (orange boxes) compared to total product per lane produced by RT-PCR as determined through quantification of fluorescence. Unpaired, two-tailed parametric *t*-test. *p*-value <0.0001 indicated by ****.

Given the proximity of the proband’s variant to the splice site, we wanted to determine whether splicing of the *TGFB2* transcript was impacted. However, *TGFB2* is not highly expressed in accessible patient tissues like blood and skin (www.proteinatlas.org), so we could not assess the impact directly from patient samples using RNA sequencing or similar *in vivo* analyses. Instead, we designed a splice reporter minigene assay based on the pSpliceExpress vector system (Kishore et al., 2008). The system contains gene fragments of *TGFB2* reference sequence (REF), proband, or variants of interest (Table 1) consisting of an intron 2 fragment with 5′ end flanked by rat insulin exon (REI2), exon 3, intron 3, exon 4, intron 4, exon 5, and intron 5 fragment’s 3′ end flanked by rat insulin exon (REI3) (Figure 2A; Supplemental Table S1).

**FIGURE 3:**
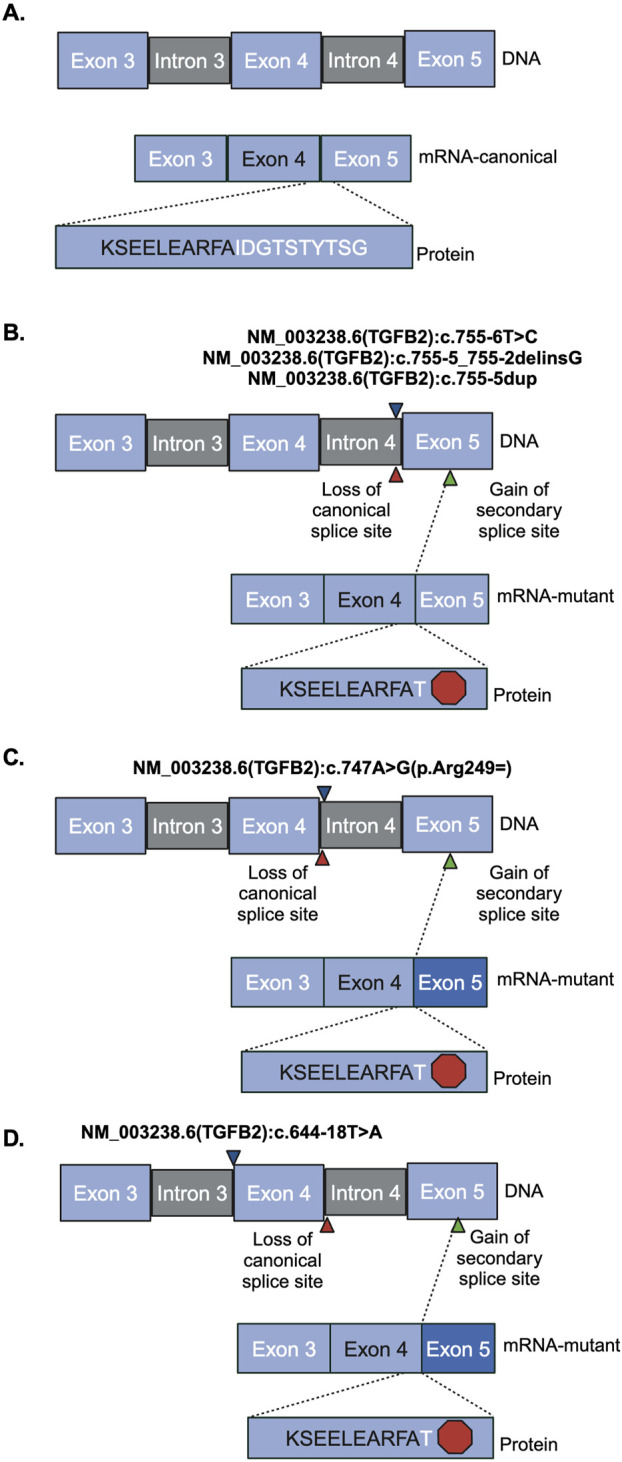
Sanger sequencing product and resulting splicing alterations. Red hexagon indicates a stop codon, blue triangles indicate where the variant is. Red triangles indicate the region of loss of canonical splice site. Green triangle indicates the region of gain of the secondary splice site. Darker blue exon indicates truncated exon. **(A)** Reference vector **(B)** NM_003238.6 (TGFB2):c.755-6T>C, NM_003238.6 (TGFB2):c.755-5_755-2delinsG, and NM_003238.6 (TGFB2):c.755-5dup **(C)** NM_003238.6 (TGFB2):c.747A>G **(D)** NM_003238.6 (TGFB2):c.644-18T>A.

The region containing these variants was inserted into the splice reporter plasmid, transfected into HEK293T cells where the artificial transcript is expressed under the control of a constitutively active promoter. RNA was extracted from cells and the resulting transcripts were analyzed using gel electrophoresis and sequencing. Amplifying the transcripts with common primers resulted in a series of products representing multiple splice products. We ran the products on an agarose gel and quantified the intensity of the bands (Figures 2B, C; Supplemental Figures S2, S3) to demonstrate that there is significant reduction in full length product between the reference vector and the proband sequence, NM_003238.6 (TGFB2):c.755-6T>C (*p* < 0.0001). We also observed reduced full length product with the variants NM_003238.6 (TGFB2):c.747A>G (*p* < 0.0001) and NM_003238.6 (TGFB2):c.644-18T>A (*p* < 0.0001). All three of these variants were classified as likely benign in ClinVar but appear to impact splicing. Sanger sequencing (Supplemental Table S2) suggests that the NM_003238.6 (TGFB2):c.755-6T>C, proband sequence, results in the use of a secondary splice acceptor 117 base pairs into exon 5. This altered transcript would result in a premature stop codon and could either lead to a truncated protein or be subject to nonsense-mediated decay (Figures 3A, B). As expected, the variant previously classified as Likely Pathogenic, NM_003238.6 (TGFB2):c.755-5_755-2delinsG, also shows no measurable level of the full length (REF) transcript. We do note that all other sequences, including the REF, show low levels of the alternate splice product. (Figures 2B, C; Supplemental Figure S2, S3).

## 4 Discussion

While there have been advances in characterizing intronic and synonymous splicing variants causative of Mendelian disease, they are still underexplored as a molecular diagnosis. In this study, we explore several variants that occur near splice junctions of *TGFB2*, a gene linked to the connective tissue disorder Loeys-Dietz Syndrome. We characterize the function of variants observed in patients and prioritize using computational predictions. Our functional data provide insight into the biological consequences of those variants. This work was motivated by the identification of an individual with features of LDS who did not have a molecular diagnosis. In fact, using current diagnostic conventions, fewer than half of individuals with LDS and other connective tissue disorders receive a molecular diagnosis and in those cases diagnosis relies on clinical features and phenotype alone (Renner et al., 2019). This limitation is not specific to LDS. Genetic diagnosis of rare disease patients is possible in approximately 50% of rare disease cases with current sequencing technologies. Continuing efforts to annotate and interpret noncoding and splicing variants combined with improved technologies for detecting repeat expansions and structural variation are slowly increasing that rate (Abul-Husn, et al., 2023; Felker, et al., 2023; Hiatt, et al., 2021).

The lack of molecular diagnosis limits a clinician’s ability to accurately diagnose patients and thus direct proper care. Here, we present a study of a unique family with both confirmed LDS and features of LDS. Several family members’ symptoms can be explained by a missense variant in *TGFBR2,* another gene associated with LDS*.* The proband in our study is a member of the same family, but lacks the established *TGFBR2* variant. Instead, the proband has a rare intronic variant in *TGFB2* (GnomAD v4.1.0 exome allele frequency 6.949e-7). This variant occurs 5 bp upstream of the splice acceptor and SpliceAI gives it a non-zero score indicating a possibility that it affects splicing. Our hypothesis is that the variants that favor the alternate acceptor reduce the level of functional protein leading to the observed phenotypes. Given the rarity of the variant, the proximity to the splice site, the SpliceAI prediction, and no other relevant variants observed; we sought to characterize the variant further. We acknowledge the statistical rarity (1 in 10, 000, 000,000) of having two independent variants in a pedigree that are causative for LDS. However, there is supporting evidence for this improbable explanation: 1) presence of only one of the two variants in affected members, 2) absence of any other candidate variants to explain the LDS phenotypes, and 3) functional data detailed in this paper.

We measured the impact on splicing of several intronic *TGFB2* variants. The SpliceAI algorithm predicted that the variant observed in our proband [NM_003238.6 (TGFB2):c.755-6T>C] and a rare variant reported in ClinVar [NM_003238.6 (TGFB2):c.644-18T>A] may interfere with canonical splicing of *TGFB2.* Both variants reduce the level of protein-generating transcript in favor of an isoform that results in a premature stop codon by formation of an alternate accepter 117 bp downstream in exon 5. This splice isoform was also observed when testing the likely pathogenic variant [NM_003238.6 (TGFB2):c.755-5_755-2delinsG]. Isoforms containing this splice junction have been previously reported (NR_138148.2, NR_138149.2), and result in nonsense-mediated decay (O’Leary et al., 2016; Strausberg et al., 2002), so it is possible that these isoforms are less common but perhaps normal splicing products of *TGFB2* expression. Other common variants in this region did not significantly alter splicing efficiency [NM_003238.6 (TGFB2):c.755-5dup and NM_003238.6 (TGFB2):c.747A>G (p.Arg249 = )]. In addition, at least one other variant impacting splicing of *TGFB2* that introduces a similar premature stop has been associated with loss of protein function (Arnaud et al., 2019). Our data support the hypothesis that the variant observed in the proband favors an isoform that creates a premature stop ultimately reducing the level of functional protein.

Our findings provide insight into the variability in phenotype and genotype in LDS, highlighting the potential importance of splicing efficiency. Given the variable expressivity and even rare incomplete penetrance observed among patients with LDS and specifically within this family it seems plausible that phenotypic differences observed between families with different disease-causing variants could partly be explained by the level of canonical transcript. This finding allows for new ACMG codes applicable to the proband in consideration of molecular studies: PM2_Supporting and PP1_Supporting (Walker et al., 2023).

This study serves the scientific and clinical communities of LDS and connective tissue disorders by contributing to the growing body of evidence supporting the relevance of intronic splicing variants in transforming growth factors and their receptors as a molecular cause for LDS (Fujiwara et al., 2018). Our work provides new evidence supporting the potential relevance of these variants to disease and demonstrates the importance of functional assays together with computational predictions to reveal the impacts that putative disease-causing variants have on phenotype. Together with clinical information, functional data and computational predictions together will contribute to improved diagnostic rates and ultimately better patient outcomes.

## Data Availability

Publicly available data was analyzed in this study. This data can be found here: ClinVar (https://www.ncbi.nlm.nih./gov/clinvar/) under the accession number SCV003221034.2. Additional datasets presented in this article are not readily available because data sharing was not consented with the patients under the IRP protocol used.
