# Supplementary material for: Novel variant alters splicing of TGFB2 in family with features of Loeys-Dietz syndrome
Source: Front Genet. 2024 Dec 16;15:1435734. doi: 10.3389/fgene.2024.1435734 (PMC11683094; doi:10.3389/fgene.2024.1435734)
Supplement: Supplementary file 1 [file DataSheet2.pdf]

Supplementery Table 2: Sanger sequencing of resolved minigene bands. Bands referenced correlate to Figure 3 labeled bands		
**Bands**	**Exons**	**Sequence**
in orange box Fig 2	3, 4, 5	ATTCTCAAGTCCAAAGATTTAACATCTCCAACCCAGCGCTACATCGACAGCAAAG<br>TTGTGAAAACAAGAGCAGAAGGCGAATGGCTCTCCTTCGATGTAACTGATGCTG<br>TTCATGAATGGCTTCACCATAAAGACAGGAACCTGGGATTTAAAATAAGCTTACA<br>CTGTCCCTGCTGCACTTTTGTACCATCTAATAATTACATCATCCCAAATAAAAGTG<br>AAGAACTAGAAGCAAGATTTGCAGGTATTGATGGCACCTCCACATATACCAGTG<br>GTGATCAGAAAACTATAAAGTCCACTAGGAAAAAAAACAGTGGGAAGACCCCAC<br>ATCTCCTGCTAGTGTTCTGGCACACCANAAAAACTTGAGTCACAACAAGACCAAC<br>CGGCGGAAGAAGCGTGCTTTGTATGCGGCCTATTGTTTT
all others indicated in Fig 2	3, 4, 117 bp into 5	ATTCTCAAGTCCAAAGATTTAACATCTCCAACCCAGCGCTACATCGACAGCAAAG<br>TTGTGAAAACAAGAGCAGAAGGCGAATGGCTCTCCTTCGATGTAACTGATGCTG<br>TTCATGAATGGCTTCACCATAAAGACAGGAACCTGGGATTTAAAATAAGCTTACA<br>CTGTCCCTGCTGCACTTTTGTACCATCTAATAATTACATCATCCCAAATAAAAGTG<br>AAGAACTAGAAGCAAGATTTGCAG
