# Supplementary material for: Novel variant alters splicing of TGFB2 in family with features of Loeys-Dietz syndrome
Source: Front Genet. 2024 Dec 16;15:1435734. doi: 10.3389/fgene.2024.1435734 (PMC11683094; doi:10.3389/fgene.2024.1435734)
Supplement: Supplementary file 2 [file Image2.pdf]

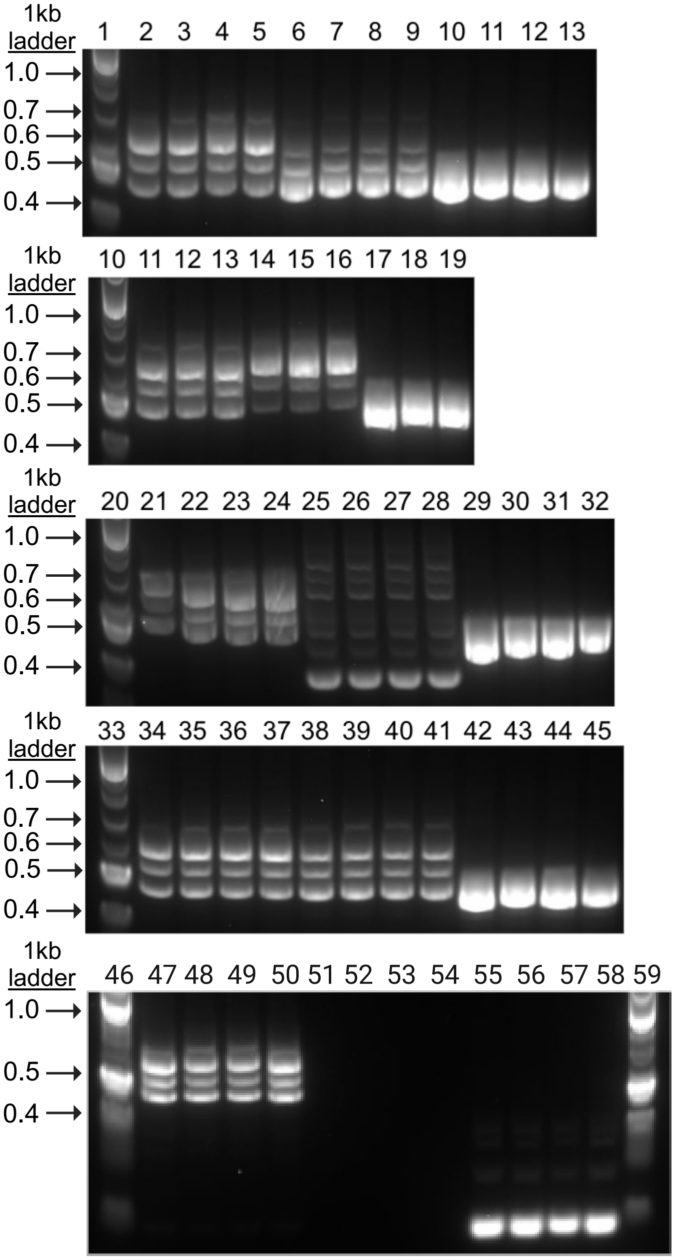

Supplemental Figure 2: Raw gel results of the RT-PCR where lanes 1, 10, 20, 33, 46 and 59 are size markers (1 kb plus ladder); 2-5, 11-13, 21-24, 34-37, and 47-55 are wild type; 6-9 are proband NM_003238.6(TGFB2):c.755-6T>C; 10-13, 17-19, 29-32, and 42-45, are likely pathogenic positive control NM_003238.6(TGFB2):c.755-5_755-2delinsG; 14-16 are a benign positive control NM_003238.6(TGFB2):c.755-5dup; 25-28 is likely benign NM_003238.6(TGFB2):c.644-18T>A; 38-41 a likely benign positive control variant NM_003238.6(TGFB2):c.747A>G; 51-54 is of the HEK293T cells; and 55-88 is of the pSpliceExpress plasmid without a minigene.
