# Supplementary material for: Novel variant alters splicing of TGFB2 in family with features of Loeys-Dietz syndrome
Source: Front Genet. 2024 Dec 16;15:1435734. doi: 10.3389/fgene.2024.1435734 (PMC11683094; doi:10.3389/fgene.2024.1435734)
Supplement: Supplementary file 3 [file DataSheet3.pdf]

Supplementary Methods

1.1 Clinical Evaluation of Connective Tissue Disorder

A standard of care process for evaluation for connective tissue disorders was followed for the clinical evaluation of the first proband (Individual II:3, Figure 1A), his two sons (Individuals III:4 and III:5, Figure 1A), and his niece (Individual III:1, Figure 1A). This included a physical exam with a medical geneticist for all individuals, a clinical connective tissue panel for the proband and his niece, targeted familial variant testing for the proband's two sons and his niece, and clinical genome sequencing for the proband's niece. When the proband was diagnosed with Loeys-Dietz syndrome, evaluation of at-risk relatives and cascade testing was recommended. His two sons then underwent a physical exam with a medical geneticist and, following established guidelines for the evaluation and diagnosis of Loeys-Dietz syndrome (Loeys et al., 2018), were recommended to proceed with targeted variant testing from the commercial lab.

The proband shows features of connective tissue disorder, the most common of which is Marfan Syndrome which was considered. A diagnosis of Marfan syndrome is made through established clinical criteria, which incorporate cardinal signs, systemic scoring guidelines, *FBN1* analysis, and the exclusion of variants in genes associated with other connective tissue disorders that have overlapping features (Loeys et al., 2010). Evaluation by a board certified medical geneticist ruled out Marfan syndrome and other connective tissue disorders in all reported individuals through application of the Marfan syndrome diagnostic criteria and/or clinical genetic testing including all genes currently associated with connective tissue disorders. All individuals in question were tested for *FBN1* variants as part of a panel and none were observed.

In the absence of a family history of Loeys-Dietz syndrome, a diagnosis is made based on the presence of a likely pathogenic or pathogenic variant in an associated gene and either (1) an aortic root enlargement (z-score ≥2.0) or type A dissection or (2) a combination of characteristic craniofacial, skeletal, cutaneous, and/or vascular manifestations (Loeys et al., 2018). The proband (Individual II:3, Figure 1A) was diagnosed with Loeys-Dietz syndrome based on the presence of a likely pathogenic *TGFBR2* variant, his aortic root enlargement, and exclusion of a diagnosis of overlapping clinical features.

A diagnosis can be made in at-risk relatives of a person with a clinical diagnosis of Loey-Dietz syndrome by identification of the pathogenic variant even if the other features are not yet present (Loeys et al., 2018). The proband's two sons (Individuals III:4 and III:5, Figure 1A) were therefore diagnosed with Loeys-Dietz based on the presence of the diagnostic variant identified in their father.

Given that a formal diagnosis of Loeys-Dietz syndrome is not possible without the identification of a pathogenic variant in an associated gene (Loeys et al., 2018), the proband's niece (Individual III:1, Figure 1A) and sister (Individual II:1; Figure 1A, 1D-E) received a <u>likely</u> diagnosis of Loeys-Dietz syndrome based on the described TGFB2 intronic splicing variant; combined presence of characteristic craniofacial, skeletal, and cutaneous features; and exclusion of a diagnosis of overlapping clinical features. It was confirmed by the commercial testing facility that the individual II:3 does not have the proband's variant NM_003238.6(TGFB2):c.755-6T>C.

1.2 Data Processing and Quality Control

After sequencing, reads were generated using Illumina's bcl2fastq (v2.20.0.422), and data were aligned to the Genome in a Bottle v2 masked human reference GRCh38 (Behera et al, 2023; https://ftp-trace.ncbi.nlm.nih.gov/ReferenceSamples/giab/release/references/GRCh38/GCA_000001405.15_GRCh38_no_alt_analysis_set_maskedGRC_exclusions.fasta.gz) using the sentieon v202112.01 implementation of BWA-mem with the -M and -K 10000000 command line options [Kendig, 2019]. Fragments mapping to multiple regions of the reference genome were removed from the analysis, as were fragments having a low-quality score. Duplicate fragments were removed using sentieon v202112.01 LocusCollector and sentieon Dedup with the --rmdup option. Variant calling then proceeded using unique, quality mappings with sentieon v202112.01 DNAscope with Illumina whole genome machine learning model v1.0 using the --pcr_indel_model none flag and known variant sites from dbsnp v146 (Freed et al, 2022). The variant's quality was assessed with sentieon DNAModelApply and variants rejected by the DNAScope model were tagged with the MLrejected flag and then removed from the analysis using bcftools v1.15.1. Variants outside of the primary chromosomes (chr1-22, chrX, chrY, chrM) were removed from the analysis using bcftools. Quality metrics were evaluated at each sample preparation and analysis stage to ensure quality data. Samples were typically required to have >= 85% bases at Q30; mean Q score >= 35.7, >98.7% reads aligned, and mean coverage >= 28x.

1.3 Genomic Analysis

Within Codicem all sequence variants were annotated with relevant reference information from established data sources to provide support for the interpretations. Variants were filtered for a total read depth of >= to 8, with at least 15% of reads supporting the alternative allele. Prospective variants were subsequently filtered to include those within a gene associated with mendelian disease in OMIM or ClinVar, further restricted to those disease genes associated with the HPO terms in the proband (generated from an in-house gene-disease annotator: https://github.com/HudsonAlpha/LayeredGraph). Variants were also restricted to rare variants (generally gnomAD genome AF <= 0.01 and exome AF <=0.005) (Karczewski et al., 2020), and a HudsonAlpha internal allele count of less than 25. Variant submissions with the assertion of pathogenic or likely pathogenic in ClinVar were also considered in specific contexts. Variants were further curated using predicted biological consequence and CADD score.

1.4 pSpliceExpress Mini Gene Splice Reporter System Additional Details

Methods previously established by (Kishore et al., 2010) to conduct a splice reporter assay but portions are elaborated on below:

gBlocks were cloned into the pSpliceExpress vector (Addgene #32485) using the NEB Gibson Assembly Master Mix (#E2611L) in a 5 µl reaction containing: 30 fmol gBlock, 25 fmol vector, 1 µl 5x BP Clonase, and molecular biology grade water up to 5 µl. This was incubated overnight (20-24 hours) at 25°C in a thermocycler. BP was inactivated for 10 minutes at 37°C with 1 µl of Proteinase K (2mg/ml stock). Using One Shot OmniMax 2-T1^{R} chemically competent cells (ThermoFisher #C854003) cells were transformed in a ratio of 2 µl DNA to 25 µl cells using standard methods otherwise.
