# Supplementary material for: Novel variant alters splicing of TGFB2 in family with features of Loeys-Dietz syndrome
Source: Front Genet. 2024 Dec 16;15:1435734. doi: 10.3389/fgene.2024.1435734 (PMC11683094; doi:10.3389/fgene.2024.1435734)
Supplement: Supplementary file 4 [file Image3.pdf]

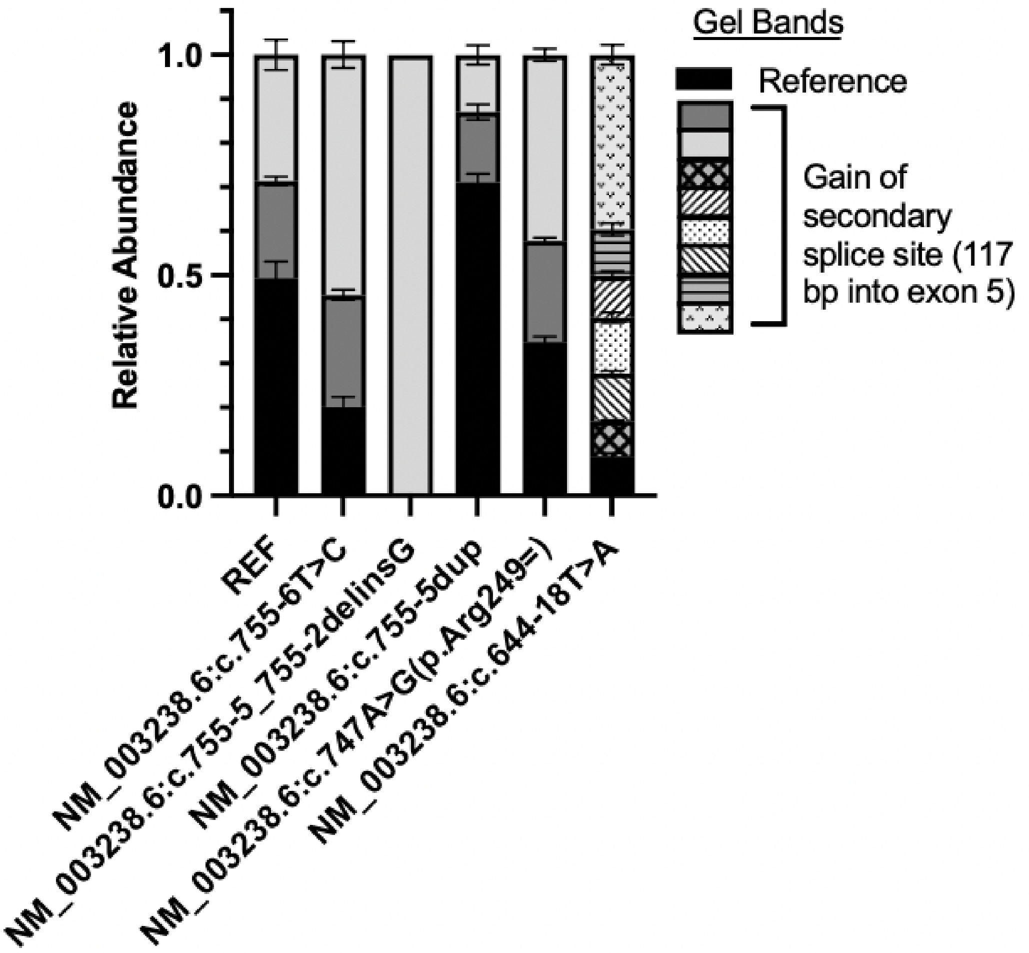

Supplemental Figure 3: Stacked barplot showing the quantified relative abundance of each observed splice product as a proportion of the total of the bands observed on the agarose gels (Fig 2B or Supplemental Fig 2) Individual bars represent each band quantified and correlating product.
