# Supplementary material for: Novel variant alters splicing of TGFB2 in family with features of Loeys-Dietz syndrome
Source: Front Genet. 2024 Dec 16;15:1435734. doi: 10.3389/fgene.2024.1435734 (PMC11683094; doi:10.3389/fgene.2024.1435734)
Supplement: Supplementary file 5 [file DataSheet1.pdf]

SupplementeryTable 1: Mini gene fragments cloned into pSpliceExpress	
**Variant**	**Sequence of gBlocks HiFi Gene Fragments**
Wild type reference	ACCGAGTTGCCCGTTAAAGTGGGGACAAGTTTGTACAAAAAAGCAGGCTGATTTAGTAGCTATTTTTTCATGGCAAATAGCCTGGTGTTGTGTTTTTATGCATGTGACCATGCAATTGAGATGACAATGCATGGCTATACTACAGTAGAGCTAAATTTAGGTAATGAATTAGAACACT<br>GTTAATAGTTTTGGTTTAGTCATGCTGTCAGAATGCCAACTCAGCCTTTTCTCTTGCTCTTTTTCCCCTCCAGATTCTCAAGTCCAAAGATTTAACATCTCCAACCCAGCGCTACATCGACAGCAAAGTTGTGAAAACAAGAGCAGAAGGCGAATGGCTCTCCTTCGATGTAACTGATG<br>CTGTTCATGAATGGCTTCACCATAAAGGTTACAAGCCACTCTCTCTTTTCCTCCCAAGATGTTCAGTATCCCTAAGTTACTTTAAATTGATTGCAGATTTAAGGGTATAGACACACATACAAATGACCTCCTTGACTTAATGTTTTCCAGACAGGAACCTGGGATTTAAAATAAGCTTACA<br>CTGTCCCTGCTGCACTTTTGTACCATCTAATAATTACATCATCCCAAATAAAAGTGAAGAACTAGAAGCAAGATTTGCAGGTAACCAAAACTTGGTCATATGAGGTGGGGAGGGAAGGGTCTATATTGATAATCTCATGGGGGATAAAATCAGTTTGCATGTGAAAATGAGACTGG<br>TAAGGCCTGGGGAGTTTCATTCATTTGGACAGTGATATGGTTGGATATACATATGTGAAGGGAGAAAGGATTGGAAGAACGGAATAGGTTCCCAGTTTATAATTCTCAATGCCCAGTGATGACTTGGTCAATAATAACTGATTAACTCATTAACCCTCCAGTAGTAAGTTAAGGCCTT<br>TGTTCTTCATTGTAGCAAACCAACTTTCCATCTATACTGATATTATAGAGAAAGATAGAATGTGTTTAAGAAAATTCATTGATAATCCCCCAAACCAAATGTCAGGAACTGACAACCGTATTTCACAGAAGAAAGGAGTCTCCTCCTGCTACAGCTTCCATAACTAACCTTCCATGTATA<br>AGAACATTCCACCTTTTCTAGTCCATACCCAGTATTTCAAGTGGGTTCCTTATCAGAATTTTATATTAGTTTATAAACACATAAAAATGTTTTGGCTTTCCCCACGCTTACCTTTAAGTAAGTTGGACAAAAGTCATGGAGAGTGAGGCGATACCAAGGGCTCAAGTCACTTCATGTATTA<br>GATGATGAGTTGTTGGATGATGAGAGGTGAGAGGAAATCCATGCCTATGTGCCGATACAGCATCTGCCCACAATGCCAGGCCAGAGAATTGATTTCCCTGCCCTCCTAACATCACCCAGGACCTCCATCCACTTTTAGAAGACTACAGCAGCCCTTGCTCATGGAAAAATACTCCCA<br>GAGGTCTAGAGATTTATTTCAGTCTCTAACCTAGTCATGATTCTCTGGAGTGTGGAGATTAAATGCTTAAACTGGCAGTCTCTTGAGCATGCTTTGGGGAGCTCAAGTTTCTAAATCTCAGATGGAATCAGGAAAGTGTTGAGGATGCCATAAAATAAATGAAAAAGCCCTTTCTTCAC<br>AGCTTCCCAAAATAGCATTAGGTAAGGGTGACTTCTGTTTCACTTGAACCTAATTCACTCCAATTCAGTTAAGTCTCAGTTTATGTTCATTAGGAAAGTCTTTTGAGTTTAGTAGTCTTAGGAAGAGTGTGAACCAGCGGGTGACAAGGCATGTTCTATTGCCATGTGTTCAGTTGTTGA<br>GGGCTTGAACGCAGCCTAAACTGAGGATTGACTAAAATAAACTGCCTAGATTGCCCTTGGACTATAGGTCAAAGACTGCTTCTGTAGTGACTTTCATAACCAACAGCCCTGGCTTTGACACCGAACTGCCTTCTGACAAGAGCCCCATTTAGAAGTGTTAATTGTCACGTGGGTAGT<br>CGTAGTCCTGTTGTGCCATATCTATTTCCATGGGGAATAACTGTTGCCAGCTGATGCTGCTTTGGTGGTCATCACTGCTATTTGTGACAATATACAAAGGAAAAAAATATGGCTGACTATATTTGATGAAGGTGAAGCTAAATGTTTATTACCCAAATGCATTTTTTCAAGGTATTGATG<br>GCACCTCCACATATACCAGTGGTGATCAGAAAACTATAAAGTCCACTAGGAAAAAAAACAGTGGGAAGACCCCACATCTCCTGCTAATGTTATTGCCCTCCTACAGACTTGAGTCACAACAGACCAACCGGCGGAAGAAGCGTGCTTTGGATGCGGCCTATTGCTTTAGGTAAAGG<br>AAAGAAAAGTAAAACCAAGTAATTGCATCTGTTAACTCTTAAACTGCCTTTGCCCTTTCTTTTACTGTGTATTGACCCAAGTGGAACTCACTGCTAAGGCCAGATAGAGTGCAGTACAGCTCCTGTGTCTCATAATACCATTCTTCCCAGAGGCTTATATTCATCAGGTCTTTAGCAGTG<br>AACCAAGTGTGAACCCAGCTTTCTTGTACAAAGTGGTCCCCAATGGCGTCCAGTTGTCACA
NM_003238.6(TGFB2):c.755-6T>C	ACCGAGTTGCCCGTTAAAGTGGGGACAAGTTTGTACAAAAAAGCAGGCTGATTTAGTAGCTATTTTTTCATGGCAAATAGCCTGGTGTTGTGTTTTTATGCATGTGACCATGCAATTGAGATGACAATGCATGGCTATACTACAGTAGAGCTAAATTTAGGTAATGAATTAGAACACT<br>GTTAATAGTTTTGGTTTAGTCATGCTGTCAGAATGCCAACTCAGCCTTTTCTCTTGCTCTTTTTCCCCTCCAGATTCTCAAGTCCAAAGATTTAACATCTCCAACCCAGCGCTACATCGACAGCAAAGTTGTGAAAACAAGAGCAGAAGGCGAATGGCTCTCCTTCGATGTAACTGATG<br>CTGTTCATGAATGGCTTCACCATAAAGGTTACAAGCCACTCTCTCTTTTCCTCCCAAGATGTTCAGTATCCCTAAGTTACTTTAAATTGATTGCAGATTTAAGGGTATAGACACACATACAAATGACCTCCTTGACTTAATGTTTTCCAGACAGGAACCTGGGATTTAAAATAAGCTTACA<br>CTGTCCCTGCTGCACTTTTGTACCATCTAATAATTACATCATCCCAAATAAAAGTGAAGAACTAGAAGCAAGATTTGCAGGTAACCAAAACTTGGTCATATGAGGTGGGGAGGGAAGGGTCTATATTGATAATCTCATGGGGGATAAAATCAGTTTGCATGTGAAAATGAGACTGG<br>TAAGGCCTGGGGAGTTTCATTCATTTGGACAGTGATATGGTTGGATATACATATGTGAAGGGAGAAAGGATTGGAAGAACGGAATAGGTTCCCAGTTTATAATTCTCAATGCCCAGTGATGACTTGGTCAATAATAACTGATTAACTCATTAACCCTCCAGTAGTAAGTTAAGGCCTT<br>TGTTCTTCATTGTAGCAAACCAACTTTCCATCTATACTGATATTATAGAGAAAGATAGAATGTGTTTAAGAAAATTCATTGATAATCCCCCAAACCAAATGTCAGGAACTGACAACCGTATTTCACAGAAGAAAGGAGTCTCCTCCTGCTACAGCTTCCATAACTAACCTTCCATGTATA<br>AGAACATTCCACCTTTTCTAGTCCATACCCAGTATTTCAAGTGGGTTCCTTATCAGAATTTTATATTAGTTTATAAACACATAAAAATGTTTTGGCTTTCCCCACGCTTACCTTTAAGTAAGTTGGACAAAAGTCATGGAGAGTGAGGCGATACCAAGGGCTCAAGTCACTTCATGTATTA<br>GATGATGAGTTGTTGGATGATGAGAGGTGAGAGGAAATCCATGCCTATGTGCCGATACAGCATCTGCCCACAATGCCAGGCCAGAGAATTGATTTCCCTGCCCTCCTAACATCACCCAGGACCTCCATCCACTTTTAGAAGACTACAGCAGCCCTTGCTCATGGAAAAATACTCCCA<br>GAGGTCTAGAGATTTATTTCAGTCTCTAACCTAGTCATGATTCTCTGGAGTGTGGAGATTAAATGCTTAAACTGGCAGTCTCTTGAGCATGCTTTGGGGAGCTCAAGTTTCTAAATCTCAGATGGAATCAGGAAAGTGTTGAGGATGCCATAAAATAAATGAAAAAGCCCTTTCTTCAC<br>AGCTTCCCAAAATAGCATTAGGTAAGGGTGACTTCTGTTTCACTTGAACCTAATTCACTCCAATTCAGTTAAGTCTCAGTTTATGTTCATTAGGAAAGTCTTTTGAGTTTAGTAGTCTTAGGAAGAGTGTGAACCAGCGGGTGACAAGGCATGTTCTATTGCCATGTGTTCAGTTGTTGA<br>GGGCTTGAACGCAGCCTAAACTGAGGATTGACTAAAATAAACTGCCTAGATTGCCCTTGGACTATAGGTCAAAGACTGCTTCTGTAGTGACTTTCATAACCAACAGCCCTGGCTTTGACACCGAACTGCCTTCTGACAAGAGCCCCATTTAGAAGTGTTAATTGTCACGTGGGTAGT<br>CGTAGTCCTGTTGTGCCATATCTATTTCCATGGGGAATAACTGTTGCCAGCTGATGCTGCTTTGGTGGTCATCACTGCTATTTGTGACAATATACAAAGGAAAAAAATATGGCTGACTATATTTGATGAAGGTGAAGCTAAATGTTTATTACCCAAATGCATTTTTTCAAGGTATTGATG<br>GCACCTCCACATATACCAGTGGTGATCAGAAAACTATAAAGTCCACTAGGAAAAAAAACAGTGGGAAGACCCCACATCTCCTGCTAATGTTATTGCCCTCCTACAGACTTGAGTCACAACAGACCAACCGGCGGAAGAAGCGTGCTTTGGATGCGGCCTATTGCTTTAGGTAAAGG<br>AAAGAAAAGTAAAACCAAGTAATTGCATCTGTTAACTCTTAAACTGCCTTTGCCCTTTCTTTTACTGTGTATTGACCCAAGTGGAACTCACTGCTAAGGCCAGATAGAGTGCAGTACAGCTCCTGTGTCTCATAATACCATTCTTCCCAGAGGCTTATATTCATCAGGTCTTTAGCAGTG<br>AACCAAGTGTGAACCCAGCTTTCTTGTACAAAGTGGTCCCCAATGGCGTCCAGTTGTCACA
NM_003238.6(TGFB2):c.755-5_755-2delinsG	ACCGAGTTGCCCGTTAAAGTGGGGACAAGTTTGTACAAAAAAGCAGGCTGATTTAGTAGCTATTTTTTCATGGCAAATAGCCTGGTGTTGTGTTTTTATGCATGTGACCATGCAATTGAGATGACAATGCATGGCTATACTACAGTAGAGCTAAATTTAGGTAATGAATTAGAACACT<br>GTTAATAGTTTTGGTTTAGTCATGCTGTCAGAATGCCAACTCAGCCTTTTCTCTTGCTCTTTTTCCCCTCCAGATTCTCAAGTCCAAAGATTTAACATCTCCAACCCAGCGCTACATCGACAGCAAAGTTGTGAAAACAAGAGCAGAAGGCGAATGGCTCTCCTTCGATGTAACTGATG<br>CTGTTCATGAATGGCTTCACCATAAAGGTTACAAGCCACTCTCTCTTTTCCTCCCAAGATGTTCAGTATCCCTAAGTTACTTTAAATTGATTGCAGATTTAAGGGTATAGACACACATACAAATGACCTCCTTGACTTAATGTTTTCCAGACAGGAACCTGGGATTTAAAATAAGCTTACA<br>CTGTCCCTGCTGCACTTTTGTACCATCTAATAATTACATCATCCCAAATAAAAGTGAAGAACTAGAAGCAAGATTTGCAGGTAACCAAAACTTGGTCATATGAGGTGGGGAGGGAAGGGTCTATATTGATAATCTCATGGGGGATAAAATCAGTTTGCATGTGAAAATGAGACTGG<br>TAAGGCCTGGGGAGTTTCATTCATTTGGACAGTGATATGGTTGGATATACATATGTGAAGGGAGAAAGGATTGGAAGAACGGAATAGGTTCCCAGTTTATAATTCTCAATGCCCAGTGATGACTTGGTCAATAATAACTGATTAACTCATTAACCCTCCAGTAGTAAGTTAAGGCCTT<br>TGTTCTTCATTGTAGCAAACCAACTTTCCATCTATACTGATATTATAGAGAAAGATAGAATGTGTTTAAGAAAATTCATTGATAATCCCCCAAACCAAATGTCAGGAACTGACAACCGTATTTCACAGAAGAAAGGAGTCTCCTCCTGCTACAGCTTCCATAACTAACCTTCCATGTATA<br>AGAACATTCCACCTTTTCTAGTCCATACCCAGTATTTCAAGTGGGTTCCTTATCAGAATTTTATATTAGTTTATAAACACATAAAAATGTTTTGGCTTTCCCCACGCTTACCTTTAAGTAAGTTGGACAAAAGTCATGGAGAGTGAGGCGATACCAAGGGCTCAAGTCACTTCATGTATTA<br>GATGATGAGTTGTTGGATGATGAGAGGTGAGAGGAAATCCATGCCTATGTGCCGATACAGCATCTGCCCACAATGCCAGGCCAGAGAATTGATTTCCCTGCCCTCCTAACATCACCCAGGACCTCCATCCACTTTTAGAAGACTACAGCAGCCCTTGCTCATGGAAAAATACTCCCA<br>GAGGTCTAGAGATTTATTTCAGTCTCTAACCTAGTCATGATTCTCTGGAGTGTGGAGATTAAATGCTTAAACTGGCAGTCTCTTGAGCATGCTTTGGGGAGCTCAAGTTTCTAAATCTCAGATGGAATCAGGAAAGTGTTGAGGATGCCATAAAATAAATGAAAAAGCCCTTTCTTCAC<br>AGCTTCCCAAAATAGCATTAGGTAAGGGTGACTTCTGTTTCACTTGAACCTAATTCACTCCAATTCAGTTAAGTCTCAGTTTATGTTCATTAGGAAAGTCTTTTGAGTTTAGTAGTCTTAGGAAGAGTGTGAACCAGCGGGTGACAAGGCATGTTCTATTGCCATGTGTTCAGTTGTTGA<br>GGGCTTGAACGCAGCCTAAACTGAGGATTGACTAAAATAAACTGCCTAGATTGCCCTTGGACTATAGGTCAAAGACTGCTTCTGTAGTGACTTTCATAACCAACAGCCCTGGCTTTGACACCGAACTGCCTTCTGACAAGAGCCCCATTTAGAAGTGTTAATTGTCACGTGGGTAGT<br>CGTAGTCCTGTTGTGCCATATCTATTTCCATGGGGAATAACTGTTGCCAGCTGATGCTGCTTTGGTGGTCATCACTGCTATTTGTGACAATATACAAAGGAAAAAAATATGGCTGACTATATTTGATGAAGGTGAAGCTAAATGTTTATTACCCAAATGCATTTTTTCAAGGTATTGATG<br>GCACCTCCACATATACCAGTGGTGATCAGAAAACTATAAAGTCCACTAGGAAAAAAAACAGTGGGAAGACCCCACATCTCCTGCTAATGTTATTGCCCTCCTACAGACTTGAGTCACAACAGACCAACCGGCGGAAGAAGCGTGCTTTGGATGCGGCCTATTGCTTTAGGTAAAGG<br>AAAGAAAAGTAAAACCAAGTAATTGCATCTGTTAACTCTTAAACTGCCTTTGCCCTTTCTTTTACTGTGTATTGACCCAAGTGGAACTCACTGCTAAGGCCAGATAGAGTGCAGTACAGCTCCTGTGTCTCATAATACCATTCTTCCCAGAGGCTTATATTCATCAGGTCTTTAGCAGTG<br>AACCAAGTGTGAACCCAGCTTTCTTGTACAAAGTGGTCCCCAATGGCGTCCAGTTGTCACA
NM_003238.6(TGFB2):c.755-5dup	ACCGAGTTGCCCGTTAAAGTGGGGACAAGTTTGTACAAAAAAGCAGGCTGATTTAGTAGCTATTTTTTCATGGCAAATAGCCTGGTGTTGTGTTTTTATGCATGTGACCATGCAATTGAGATGACAATGCATGGCTATACTACAGTAGAGCTAAATTTAGGTAATGAATTAGAACACT<br>GTTAATAGTTTTGGTTTAGTCATGCTGTCAGAATGCCAACTCAGCCTTTTCTCTTGCTCTTTTTCCCCTCCAGATTCTCAAGTCCAAAGATTTAACATCTCCAACCCAGCGCTACATCGACAGCAAAGTTGTGAAAACAAGAGCAGAAGGCGAATGGCTCTCCTTCGATGTAACTGATG<br>CTGTTCATGAATGGCTTCACCATAAAGGTTACAAGCCACTCTCTCTTTTCCTCCCAAGATGTTCAGTATCCCTAAGTTACTTTAAATTGATTGCAGATTTAAGGGTATAGACACACATACAAATGACCTCCTTGACTTAATGTTTTCCAGACAGGAACCTGGGATTTAAAATAAGCTTACA<br>CTGTCCCTGCTGCACTTTTGTACCATCTAATAATTACATCATCCCAAATAAAAGTGAAGAACTAGAAGCAAGATTTGCAGGTAACCAAAACTTGGTCATATGAGGTGGGGAGGGAAGGGTCTATATTGATAATCTCATGGGGGATAAAATCAGTTTGCATGTGAAAATGAGACTGG<br>TAAGGCCTGGGGAGTTTCATTCATTTGGACAGTGATATGGTTGGATATACATATGTGAAGGGAGAAAGGATTGGAAGAACGGAATAGGTTCCCAGTTTATAATTCTCAATGCCCAGTGATGACTTGGTCAATAATAACTGATTAACTCATTAACCCTCCAGTAGTAAGTTAAGGCCTT<br>TGTTCTTCATTGTAGCAAACCAACTTTCCATCTATACTGATATTATAGAGAAAGATAGAATGTGTTTAAGAAAATTCATTGATAATCCCCCAAACCAAATGTCAGGAACTGACAACCGTATTTCACAGAAGAAAGGAGTCTCCTCCTGCTACAGCTTCCATAACTAACCTTCCATGTATA<br>AGAACATTCCACCTTTTCTAGTCCATACCCAGTATTTCAAGTGGGTTCCTTATCAGAATTTTATATTAGTTTATAAACACATAAAAATGTTTTGGCTTTCCCCACGCTTACCTTTAAGTAAGTTGGACAAAAGTCATGGAGAGTGAGGCGATACCAAGGGCTCAAGTCACTTCATGTATTA<br>GATGATGAGTTGTTGGATGATGAGAGGTGAGAGGAAATCCATGCCTATGTGCCGATACAGCATCTGCCCACAATGCCAGGCCAGAGAATTGATTTCCCTGCCCTCCTAACATCACCCAGGACCTCCATCCACTTTTAGAAGACTACAGCAGCCCTTGCTCATGGAAAAATACTCCCA<br>GAGGTCTAGAGATTTATTTCAGTCTCTAACCTAGTCATGATTCTCTGGAGTGTGGAGATTAAATGCTTAAACTGGCAGTCTCTTGAGCATGCTTTGGGGAGCTCAAGTTTCTAAATCTCAGATGGAATCAGGAAAGTGTTGAGGATGCCATAAAATAAATGAAAAAGCCCTTTCTTCAC<br>AGCTTCCCAAAATAGCATTAGGTAAGGGTGACTTCTGTTTCACTTGAACCTAATTCACTCCAATTCAGTTAAGTCTCAGTTTATGTTCATTAGGAAAGTCTTTTGAGTTTAGTAGTCTTAGGAAGAGTGTGAACCAGCGGGTGACAAGGCATGTTCTATTGCCATGTGTTCAGTTGTTGA<br>GGGCTTGAACGCAGCCTAAACTGAGGATTGACTAAAATAAACTGCCTAGATTGCCCTTGGACTATAGGTCAAAGACTGCTTCTGTAGTGACTTTCATAACCAACAGCCCTGGCTTTGACACCGAACTGCCTTCTGACAAGAGCCCCATTTAGAAGTGTTAATTGTCACGTGGGTAGT<br>CGTAGTCCTGTTGTGCCATATCTATTTCCATGGGGAATAACTGTTGCCAGCTGATGCTGCTTTGGTGGTCATCACTGCTATTTGTGACAATATACAAAGGAAAAAAATATGGCTGACTATATTTGATGAAGGTGAAGCTAAATGTTTATTACCCAAATGCATTTTTTCAAGGTATTGATG<br>GCACCTCCACATATACCAGTGGTGATCAGAAAACTATAAAGTCCACTAGGAAAAAAAACAGTGGGAAGACCCCACATCTCCTGCTAATGTTATTGCCCTCCTACAGACTTGAGTCACAACAGACCAACCGGCGGAAGAAGCGTGCTTTGGATGCGGCCTATTGCTTTAGGTAAAGG<br>AAAGAAAAGTAAAACCAAGTAATTGCATCTGTTAACTCTTAAACTGCCTTTGCCCTTTCTTTTACTGTGTATTGACCCAAGTGGAACTCACTGCTAAGGCCAGATAGAGTGCAGTACAGCTCCTGTGTCTCATAATACCATTCTTCCCAGAGGCTTATATTCATCAGGTCTTTAGCAGTG<br>AACCAAGTGTGAACCCAGCTTTCTTGTACAAAGTGGTCCCCAATGGCGTCCAGTTGTCACA
NM_003238.6(TGFB2):c.747A>G(p.Arg249=)	ACCGAGTTGCCCGTTAAAGTGGGGACAAGTTTGTACAAAAAAGCAGGCTGATTTAGTAGCTATTTTTTCATGGCAAATAGCCTGGTGTTGTGTTTTTATGCATGTGACCATGCAATTGAGATGACAATGCATGGCTATACTACAGTAGAGCTAAATTTAGGTAATGAATTAGAACACT<br>GTTAATAGTTTTGGTTTAGTCATGCTGTCAGAATGCCAACTCAGCCTTTTCTCTTGCTCTTTTTCCCCTCCAGATTCTCAAGTCCAAAGATTTAACATCTCCAACCCAGCGCTACATCGACAGCAAAGTTGTGAAAACAAGAGCAGAAGGCGAATGGCTCTCCTTCGATGTAACTGATG<br>CTGTTCATGAATGGCTTCACCATAAAGGTTACAAGCCACTCTCTCTTTTCCTCCCAAGATGTTCAGTATCCCTAAGTTACTTTAAATTGATTGCAGATTTAAGGGTATAGACACACATACAAATGACCTCCTTGACTTAATGTTTTCCAGACAGGAACCTGGGATTTAAAATAAGCTTACA<br>CTGTCCCTGCTGCACTTTTGTACCATCTAATAATTACATCATCCCAAATAAAAGTGAAGAACTAGAAGCAAGATTTGCAGGTAACCAAAACTTGGTCATATGAGGTGGGGAGGGAAGGGTCTATATTGATAATCTCATGGGGGATAAAATCAGTTTGCATGTGAAAATGAGACTGG<br>TAAGGCCTGGGGAGTTTCATTCATTTGGACAGTGATATGGTTGGATATACATATGTGAAGGGAGAAAGGATTGGAAGAACGGAATAGGTTCCCAGTTTATAATTCTCAATGCCCAGTGATGACTTGGTCAATAATAACTGATTAACTCATTAACCCTCCAGTAGTAAGTTAAGGCCTT<br>TGTTCTTCATTGTAGCAAACCAACTTTCCATCTATACTGATATTATAGAGAAAGATAGAATGTGTTTAAGAAAATTCATTGATAATCCCCCAAACCAAATGTCAGGAACTGACAACCGTATTTCACAGAAGAAAGGAGTCTCCTCCTGCTACAGCTTCCATAACTAACCTTCCATGTATA<br>AGAACATTCCACCTTTTCTAGTCCATACCCAGTATTTCAAGTGGGTTCCTTATCAGAATTTTATATTAGTTTATAAACACATAAAAATGTTTTGGCTTTCCCCACGCTTACCTTTAAGTAAGTTGGACAAAAGTCATGGAGAGTGAGGCGATACCAAGGGCTCAAGTCACTTCATGTATTA<br>GATGATGAGTTGTTGGATGATGAGAGGTGAGAGGAAATCCATGCCTATGTGCCGATACAGCATCTGCCCACAATGCCAGGCCAGAGAATTGATTTCCCTGCCCTCCTAACATCACCCAGGACCTCCATCCACTTTTAGAAGACTACAGCAGCCCTTGCTCATGGAAAAATACTCCCA<br>GAGGTCTAGAGATTTATTTCAGTCTCTAACCTAGTCATGATTCTCTGGAGTGTGGAGATTAAATGCTTAAACTGGCAGTCTCTTGAGCATGCTTTGGGGAGCTCAAGTTTCTAAATCTCAGATGGAATCAGGAAAGTGTTGAGGATGCCATAAAATAAATGAAAAAGCCCTTTCTTCAC<br>AGCTTCCCAAAATAGCATTAGGTAAGGGTGACTTCTGTTTCACTTGAACCTAATTCACTCCAATTCAGTTAAGTCTCAGTTTATGTTCATTAGGAAAGTCTTTTGAGTTTAGTAGTCTTAGGAAGAGTGTGAACCAGCGGGTGACAAGGCATGTTCTATTGCCATGTGTTCAGTTGTTGA<br>GGGCTTGAACGCAGCCTAAACTGAGGATTGACTAAAATAAACTGCCTAGATTGCCCTTGGACTATAGGTCAAAGACTGCTTCTGTAGTGACTTTCATAACCAACAGCCCTGGCTTTGACACCGAACTGCCTTCTGACAAGAGCCCCATTTAGAAGTGTTAATTGTCACGTGGGTAGT<br>CGTAGTCCTGTTGTGCCATATCTATTTCCATGGGGAATAACTGTTGCCAGCTGATGCTGCTTTGGTGGTCATCACTGCTATTTGTGACAATATACAAAGGAAAAAAATATGGCTGACTATATTTGATGAAGGTGAAGCTAAATGTTTATTACCCAAATGCATTTTTTCAAGGTATTGATG<br>GCACCTCCACATATACCAGTGGTGATCAGAAAACTATAAAGTCCACTAGGAAAAAAAACAGTGGGAAGACCCCACATCTCCTGCTAATGTTATTGCCCTCCTACAGACTTGAGTCACAACAGACCAACCGGCGGAAGAAGCGTGCTTTGGATGCGGCCTATTGCTTTAGGTAAAGG<br>AAAGAAAAGTAAAACCAAGTAATTGCATCTGTTAACTCTTAAACTGCCTTTGCCCTTTCTTTTACTGTGTATTGACCCAAGTGGAACTCACTGCTAAGGCCAGATAGAGTGCAGTACAGCTCCTGTGTCTCATAATACCATTCTTCCCAGAGGCTTATATTCATCAGGTCTTTAGCAGTG<br>AACCAAGTGTGAACCCAGCTTTCTTGTACAAAGTGGTCCCCAATGGCGTCCAGTTGTCACA
NM_003238.6(TGFB2):c.644-18T>A	ACCGAGTTGCCCGTTAAAGTGGGGACAAGTTTGTACAAAAAAGCAGGCTGATTTAGTAGCTATTTTTTCATGGCAAATAGCCTGGTGTTGTGTTTTTATGCATGTGACCATGCAATTGAGATGACAATGCATGGCTATACTACAGTAGAGCTAAATTTAGGTAATGAATTAGAACACT<br>GTTAATAGTTTTGGTTTAGTCATGCTGTCAGAATGCCAACTCAGCCTTTTCTCTTGCTCTTTTTCCCCTCCAGATTCTCAAGTCCAAAGATTTAACATCTCCAACCCAGCGCTACATCGACAGCAAAGTTGTGAAAACAAGAGCAGAAGGCGAATGGCTCTCCTTCGATGTAACTGATG<br>CTGTTCATGAATGGCTTCACCATAAAGGTTACAAGCCACTCTCTCTTTTCCTCCCAAGATGTTCAGTATCCCTAAGTTACTTTAAATTGATTGCAGATTTAAGGGTATAGACACACATACAAATGACCTCCTAGACTTAATGTTTTCCAGACAGGAACCTGGGATTTAAAATAAGCTTACA<br>CTGTCCCTGCTGCACTTTTGTACCATCTAATAATTACATCATCCCAAATAAAAGTGAAGAACTAGAAGCAAGATTTGCAGGTAACCAAAACTTGGTCATATGAGGTGGGGAGGGAAGGGTCTATATTGATAATCTCATGGGGGATAAAATCAGTTTGCATGTGAAAATGAGACTGG<br>TAAGGCCTGGGGAGTTTCATTCATTTGGACAGTGATATGGTTGGATATACATATGTGAAGGGAGAAAGGATTGGAAGAACGGAATAGGTTCCCAGTTTATAATTCTCAATGCCCAGTGATGACTTGGTCAATAATAACTGATTAACTCATTAACCCTCCAGTAGTAAGTTAAGGCCTT<br>TGTTCTTCATTGTAGCAAACCAACTTTCCATCTATACTGATATTATAGAGAAAGATAGAATGTGTTTAAGAAAATTCATTGATAATCCCCCAAACCAAATGTCAGGAACTGACAACCGTATTTCACAGAAGAAAGGAGTCTCCTCCTGCTACAGCTTCCATAACTAACCTTCCATGTATA<br>AGAACATTCCACCTTTTCTAGTCCATACCCAGTATTTCAAGTGGGTTCCTTATCAGAATTTTATATTAGTTTATAAACACATAAAAATGTTTTGGCTTTCCCCACGCTTACCTTTAAGTAAGTTGGACAAAAGTCATGGAGAGTGAGGCGATACCAAGGGCTCAAGTCACTTCATGTATTA<br>GATGATGAGTTGTTGGATGATGAGAGGTGAGAGGAAATCCATGCCTATGTGCCGATACAGCATCTGCCCACAATGCCAGGCCAGAGAATTGATTTCCCTGCCCTCCTAACATCACCCAGGACCTCCATCCACTTTTAGAAGACTACAGCAGCCCTTGCTCATGGAAAAATACTCCCA<br>GAGGTCTAGAGATTTATTTCAGTCTCTAACCTAGTCATGATTCTCTGGAGTGTGGAGATTAAATGCTTAAACTGGCAGTCTCTTGAGCATGCTTTGGGGAGCTCAAGTTTCTAAATCTCAGATGGAATCAGGAAAGTGTTGAGGATGCCATAAAATAAATGAAAAAGCCCTTTCTTCAC<br>AGCTTCCCAAAATAGCATTAGGTAAGGGTGACTTCTGTTTCACTTGAACCTAATTCACTCCAATTCAGTTAAGTCTCAGTTTATGTTCATTAGGAAAGTCTTTTGAGTTTAGTAGTCTTAGGAAGAGTGTGAACCAGCGGGTGACAAGGCATGTTCTATTGCCATGTGTTCAGTTGTTGA<br>GGGCTTGAACGCAGCCTAAACTGAGGATTGACTAAAATAAACTGCCTAGATTGCCCTTGGACTATAGGTCAAAGACTGCTTCTGTAGTGACTTTCATAACCAACAGCCCTGGCTTTGACACCGAACTGCCTTCTGACAAGAGCCCCATTTAGAAGTGTTAATTGTCACGTGGGTAGT<br>CGTAGTCCTGTTGTGCCATATCTATTTCCATGGGGAATAACTGTTGCCAGCTGATGCTGCTTTGGTGGTCATCACTGCTATTTGTGACAATATACAAAGGAAAAAAATATGGCTGACTATATTTGATGAAGGTGAAGCTAAATGTTTATTACCCAAATGCATTTTTTCAAGGTATTGATG<br>GCACCTCCACATATACCAGTGGTGATCAGAAAACTATAAAGTCCACTAGGAAAAAAAACAGTGGGAAGACCCCACATCTCCTGCTAATGTTATTGCCCTCCTACAGACTTGAGTCACAACAGACCAACCGGCGGAAGAAGCGTGCTTTGGATGCGGCCTATTGCTTTAGGTAAAGG<br>AAAGAAAAGTAAAACCAAGTAATTGCATCTGTTAACTCTTAAACTGCCTTTGCCCTTTCTTTTACTGTGTATTGACCCAAGTGGAACTCACTGCTAAGGCCAGATAGAGTGCAGTACAGCTCCTGTGTCTCATAATACCATTCTTCCCAGAGGCTTATATTCATCAGGTCTTTAGCAGTG<br>AACCAAGTGTGAACCCAGCTTTCTTGTACAAAGTGGTCCCCAATGGCGTCCAGTTGTCACA
