# Supplementary material for: Novel variant alters splicing of TGFB2 in family with features of Loeys-Dietz syndrome
Source: Front Genet. 2024 Dec 16;15:1435734. doi: 10.3389/fgene.2024.1435734 (PMC11683094; doi:10.3389/fgene.2024.1435734)
Supplement: Supplementary file 6 [file Image1.pdf]

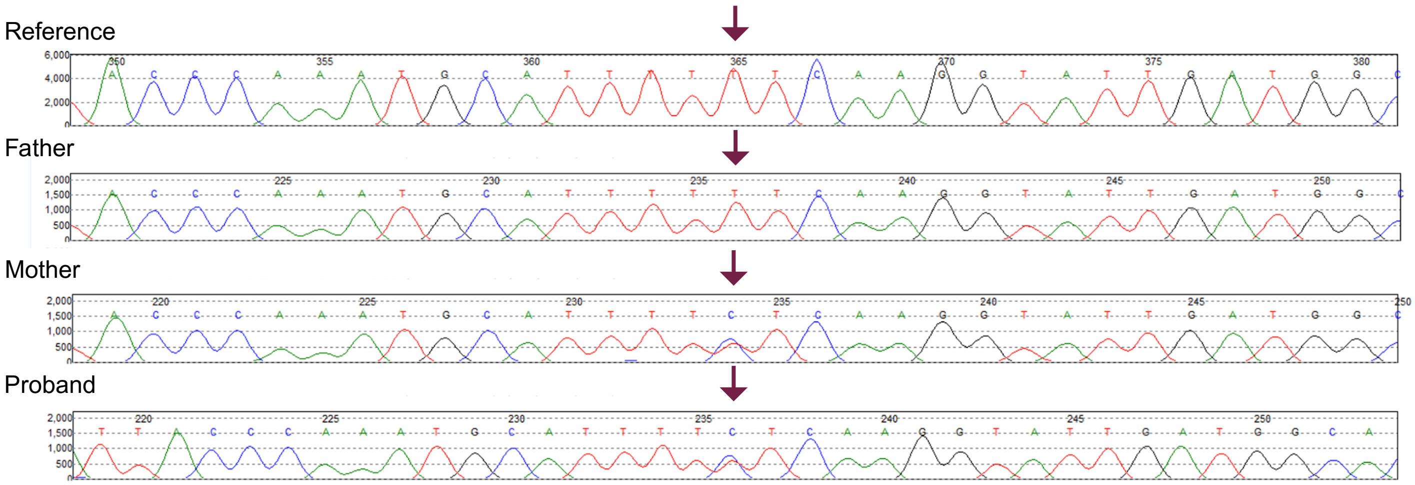

Supplemental Figure 1: *TGFB2* Sanger traces for reference, father, mother, and proband. Arrow is pointing to the NM_003238.6(TGFB2):c.755-6T>C variant found in both mother and proband.
